# Introduction of a Divergent Canine Parvovirus Type 2b Strain with a Dog in Sicily, Southern Italy, Through the Mediterranean Sea Route to Europe

**DOI:** 10.3390/pathogens14020108

**Published:** 2025-01-23

**Authors:** Francesco Mira, Giovanni Franzo, Giorgia Schirò, Domenico Vicari, Giuseppa Purpari, Vincenza Cannella, Elisabetta Giudice, Martino Trapani, Anna Carrozzo, Giada Spene, Virginia Talarico, Annalisa Guercio

**Affiliations:** 1 Istituto Zooprofilattico Sperimentale della Sicilia “A. Mirri”, 90129 Palermo, PA, Italy; francesco.mira@izssicilia.it (F.M.); domenico.vicari@izssicilia.it (D.V.); giuseppa.purpari@izssicilia.it (G.P.); vincenza.cannella@izssicilia.it (V.C.); anna.carrozzo@izssicilia.it (A.C.); spenegiada@gmail.com (G.S.); virginia.av@libero.it (V.T.); annalisa.guercio@izssicilia.it (A.G.); 2 Department of Veterinary Sciences, University of Messina, Polo Universitario dell’Annunziata, 98168 Messina, ME, Italy; elisabetta.giudice@unime.it; 3 Department of Animal Medicine, Production and Health (MAPS), Padua University, 35020 Legnaro, PD, Italy; giovanni.franzo@unipd.it; 4 Azienda Sanitaria Provinciale di Trapani, Dipartimento di Prevenzione Veterinaria, U.O.S. Igiene degli Allevamenti e delle Produzioni Zootecniche (SIAPZ) Trapani-Pantelleria, 91016 Erice, TP, Italy; martino.trapani@asptrapani.it

**Keywords:** canine parvovirus type 2, dogs, mediterranean sea, transients and migrants, northern Africa, Turkey, Europe, genomics, phylogeography

## Abstract

Despite over four decades since its emergence, canine parvovirus type 2 (CPV-2) remains a relevant disease for dogs. Few studies, primarily only recent ones based on phylodynamic and phylogeography approaches, have highlighted the impact of rapid and long-distance transport of dogs on the CPV-2 spreading dynamics. The present study reports the genomic characterization of a CPV-2 strain detected in a dog introduced into Italy from the coasts of North Africa through the Mediterranean Sea route to Europe. The nearly complete CPV-2 sequence was obtained and analyzed. The viral isolate was characterized as a CPV-2b variant, showing genetic signatures distinct from those of CPV-2 strains detected to date in Europe. Phylodynamic and phylogeographic approaches revealed a close correlation with CPV-2 strains recently reported in the Middle East (Turkey and Egypt), which likely originated or co-evolved from Asian ones. It is at least suggestive that the inferred spreading pattern overlaps with the routes often followed by migrants travelling from Asia and Middle East to Europe, passing through Africa. This evidence for the introduction of CPV-2 via the Mediterranean Sea route to Europe highlights the relevant role of the dog movements in the global spread of emerging or re-emerging viral pathogens.

## 1. Introduction

Canine parvovirus type 2 (CPV-2) is a small, non-enveloped virus, taxonomically included in the species *Protoparvovirus carnivoran1* (family *Parvoviridae*, subfamily *Parvovirinae*, and genus *Protoparvovirus*), along with closely related viruses, such as raccoon parvovirus, mink enteritis virus, and feline panleukopenia [1]. CPV-2 genome consists of an approximately 5,200 nucleotide (nt) long, single-stranded DNA molecule, containing two open reading frames (ORFs) encoding, through alternative splicing of the same mRNAs, for two non-structural (NS1–NS2) and two structural (VP1–VP2) proteins [2].

CPV-2 is considered one of the main viral canine pathogens, causing an acute and often fatal disease of domestic dogs and other carnivore domestic or wild species, characterized by severe gastroenteritis and lymphopenia, with morbidity and mortality rates [2,3].

The original type CPV-2, first identified in the late 1970s and rapidly gaining pandemic proportions in the domestic canine species, has been soon replaced by three antigenic and genetic variants, namely CPV-2a, CPV-2b, and CPV-2c [4,5,6], currently distributed worldwide, circulating with different local frequencies [7]. In Europe, CPV-2a/CPV-2b and CPV-2c variants were first described in the late 1980s and early 1990s (CPV-2a/-2b) and early 2000s (CPV-2c) [6,8,9], with different geographical and temporal frequencies during the last forty years [10]. CPV-2 has rapidly spread because of the movement of dogs, by air, land, or sea, or through fomites contaminated by faecal material [11]. Considering the Italian scenario, although with some differences on regional bases, a relatively higher prevalence of CPV-2a, followed by CPV-2b and CPV-2c (CPV-2a = 45.5%; CPV-2b = 14.65; CPV-2c = 39.8%), was observed, with the exception in the Sardinia region, where a higher prevalence of CPV-2b (80%) was identified, and Sicily region, where a higher prevalence of CPV-2c (72%) was reported [7,12,13]. These three CPV-2 variants are distinguished according to the 426-amino-acid (aa) residue of the VP2 protein (CPV-2a: Asn (2a); CPV-2b: Asp; CPV-2c: Glu) [14]. Prophylaxis of CPV infection relies mainly on extensive vaccination, by using modified live virus (MLV) vaccines [2]. Classical commercially available vaccines are prepared by using the original type CPV-2 or its CPV-2b variant [2], while more recently, recombinant ones are based on a genomic fragment encoding for the major capsid protein of the CPV-2c variant.

CPV-2 shows a high genomic substitution rate, similar to that of some RNA viruses [15], and this contributes to the occurrence of several point mutations, mostly in VP2 coding ORFs [16,17], and to the emergence of multiple lineages, with variable distribution over time and space [18,19,20,21]. The growing amount of CPV-2 complete genome and VP2 sequences has provided new insights into viral evolution and underlined how the CPV-2 typing based on VP2-426 aa residue has become less reliable for elucidating the evolutionary relationships between the circulating strains, leading to updated classifications based on their phylogenetic relationships [22].

Similarly, the growing number of CPV-2 complete genome sequences has contributed to the in-depth analysis of circulating strains, allowing the detailing of the phylodynamic features and, in some cases, to track the short- or long-distance spreading dynamics. The long-distance introduction/transport of CPV-2 or other canine pathogens has been reported [23,24,25,26] and, in some cases, the subsequent local spreading and the rapid replacement of previous circulating lineages have been also reported [7,27]. Nonetheless, there are still limited available data on the potential drivers for the emergence and spread of different CPV-2 variants to distant areas. Moreover, the dynamic international scenario, the complex network of human and animal transport, and the increasing role of dogs as companion animals in different cultures and societies have become potential critical determinants for the viral dispersal dynamics, deserving to be considered and analyzed. Aiming to contribute to this perspective, this study reports the diagnostic approach to detect and characterize selected viral pathogens in a dog introduced in Italy from the coasts of North Africa through the Mediterranean Sea route to Europe.

## 2. Materials and Methods

### 2.1. Case Description

Tested samples were collected from a four-year, female, Chihuahua breed dog that arrived on the Mediterranean coast of Sicily along with the owner, a migrant crossing the Mediterranean Sea by boats. Soon after their rescue, the dog was isolated, and it died a few hours later, showing profuse bloody diarrhea. Information relating to previous vaccinations was not clearly demonstrable, as the dog was not identified by microchip and official veterinary documents were not provided. The dog was then delivered to the Istituto Zooprofilattico Sperimentale della Sicilia “A. Mirri” (Palermo, Italy) for diagnostic purposes. The animal was then subject to a necropsy, and tissue samples (brain, lung, liver, and intestine) were collected for downstream analyses.

### 2.2. Virus Detection and Isolation

Briefly, DNA and RNA were extracted from tissue samples by using the DNeasy Blood & Tissue Kit (Qiagen S.p.A, Hilden, Germany) and QIAamp Viral RNA Mini Kit (Qiagen S.p.A., Hilden, Germany), respectively, according to the manufacturer’s instructions, and stored at −80 °C until processed.

Immunofluorescence examination of the brain sample was conducted to detect rabies virus antigen, according to the Manual of Diagnostic Tests and Vaccines for Terrestrial Animals by WOAH [28], and real-time RT-PCR assay was performed for the detection of Lyssavirus RNA [29]. Other samples (brain, liver, lung, and intestine) were then tested to detect the CPV-2 DNA and the canine coronavirus (CCoV) RNA, according to previously described molecular assays [30,31]. In more detail, CPV-2 detection was carried out using the GoTaq G2 DNA Polymerase (Promega Italia s.r.l., Milan, Italy) in a 50 μL reaction mix consisting of 5× GoTaq^®^ Reaction Buffer 1×, MgCl_2_ 0.5 mM, dNTP mix 0,2 mM, 0.5 μM of each primer VP2-850-Forward and VP2-1550-Reverse (Table 1), GoTaq^®^ G2 DNA Polymerase 1.25 U, and 5 μL of DNA extract. Amplification was conducted under the following thermal conditions: 94 °C for 2 min followed by 40 cycles at 94 °C for 30 s, 55 °C for 60 s, 72 °C for 60 s, and a final extension at 72 °C for 10 min. A field CPV-2c strain (code: VIR AR 12511 1), obtained from the Biobanca del Mediterraneo, Italy (www.bbmed.it, accessed on 12 April 2024), was used as a positive control.

To isolate the detected CPV-2 strain for future studies, the lung sample was processed accordingly and inoculated on the permissive cell lines A-72 (fibroblast-like cells derived from canine tumor), provided by the Biobank of Veterinary Resources of the Istituto Zooprofilattico Sperimentale della Lombardia e dell’Emilia Romagna (www.ibvr.org, accessed on 12 April 2024) (code: BS TCL 1). The sample was homogenized (10% w/v) in a culture medium (Eagle’s Minimum Essential Medium (EMEM); Sigma–Aldrich^®^, Milan, Italy) supplemented with an antibiotic and antimycotic solution 10× (1000 U/mL penicillin G sodium salt, 1 mg/mL streptomycin sulfate, 2,5 μg/mL amphotericin B; Euro Clone^®^, Milan, Italy). The supernatant of the tissue sample homogenate was processed according to routinary laboratory procedures as previously described [32]: briefly, the homogenate was diluted at 1:10 in EMEM supplemented with an antibiotic and antimycotic solution 1× (100 U/mL penicillin G sodium salt, 0.1 mg/mL streptomycin sulfate, and 0.25 μg/mL amphotericin B; Euro Clone^®^), and 1 mL of diluted homogenate was added to a A-72 cell monolayer (in mitotic phase; 60/70% confluent) in a T25 tissue flask. Inoculated A72 cells were incubated at 37 °C in a 5% CO_2_ incubator. After 30 min, 6 mL of EMEM supplemented with an antibiotic and antimycotic solution 1×, 1% sodium pyruvate, and 2% FBS was added, and cells were cultured at 37 °C in 5% CO_2_ for 6–7 days. A negative control (non-inoculated A72 cells) was also performed. Inoculated cells were daily monitored, and viral growth was evaluated by detection of the cytopathic effect (CPE). More blind passages were performed if the CPE was not observed. Monolayers were then subjected to three cycles of freezing and thawing, collected after centrifugation at a low speed (1500× *g* for 10 min at 4 °C) and tested for CPV-2 DNA using the previously cited PCR assay [30] to confirm the presence of the infectious virus.

### 2.3. Genetic Characterization of the CPV-2 Strain

A previously described protocol [16] using the primer pairs (Table 1) described by Pérez et al. [17] was used to amplify the nearly complete CPV-2 sequence, and the PCR products were subjected to Sanger sequencing in both directions.

Overlapping sequences were then assembled and analyzed using BioEdit ver 7.0.5.3 software [33], and sequence data were submitted to the GenBank databases under accession number PQ177900. The virus was initially typed according to the critical VP2 amino acid residues, as described by Martella et al. [14], and nucleotide sequence similarities and statistical significance of matches were inferred using the Basic Local Alignment Search Tool (BLAST) tool [34] (accessed on 25 May 2024).

### 2.4. CPV-2 Phylogenetic Analysis

VP2 and nearly complete genome *Protoparvovirus carnivoran1* sequence reference datasets were generated by searching the nucleotide BLAST (BLASTn) tool (accessed on 24 June 2024) and setting the maximum number of retrieved sequences to 5000, and related sequences were downloaded in FASTA format. Only sequences for which data on collection host, country and date were available were included in the study.

Sequences were aligned using MAFFT ver. 7.487 [35], and sequences with poor alignment quality, premature stop codon, or frameshift mutation were discarded from the analysis. To achieve a balance between sequence length and the number of included strains, sequences were trimmed to include the nucleotides 1–1746, encoding 582 amino acid residues. When complete genome analysis was performed, only sequences longer than 4269 bp were considered.

The final dataset therefore included a total number of 2712 VP2 gene sequences and 871 nearly complete genomic sequences. A phylogenetics tree was constructed with the IQ-Tree software ver. 2.3.6 [36] by selecting the best substitution model and was calculated using the same software. The inferred clades’ reliability was assessed by performing 1000 bootstrap replicates.

### 2.5. CPV-2 Phylogeographic Analysis

To reduce computational complexity and focus on reconstructing the final stage of the migratory process for the strain under consideration, rather than replicating the overall dispersal pattern already investigated in previous studies, a subset of partial VP2 sequences, determined through phylogenetic analysis, was selected and analyzed using the Bayesian serial coalescent approach implemented in BEAST 1.10 [37]. The nucleotide substitution model was determined based on the BIC score calculated using JmodelTest [38].

The molecular clock model was chosen by calculating the marginal likelihood estimation through path-sampling and stepping-stone methods, as recommended by Baele et al. [39]. To account for the viral population dynamics (relative genetic diversity: effective population size × generation time; Ne × τ) variation over time, the non-parametric Bayesian Skygrid method [40] was applied.

Additionally, a discrete-state phylogeographic analysis was performed on the VP2 dataset following the methodology of Lemey et al., 2009 [41]. An asymmetric migration model incorporating Bayesian stochastic search variable selection (BSSVS) was implemented to identify the most parsimonious description of the spreading process and calculate Bayes factor (BF) values, indicating the statistical significance of inferred migration pathways between regions.

Each analysis involved an independent run of 200 million generations. Results were examined using Tracer 1.7 after discarding the initial 20% as the burn-in period. Only results with an estimated sample size (ESS) above 200 and adequate convergence and mixing were considered reliable. Parameter estimates were summarized as means with 95% highest posterior density (95HPD) intervals. Maximum clade credibility (MCC) trees were constructed and annotated using TreeAnnotator from the BEAST package ver. 1.10.4. The BF associated to each between-country connection was calculated using SPREAD4 (www.spreadviz.org, accessed on 4 November 2024) [42] and considered statistically significant when greater than 10.

## 3. Results

### 3.1. Virus Detection and Isolation

Preliminary assays targeting the rabies virus antigen and *Lyssavirus* RNA revealed negative results, excluding the presence of these viruses. All samples (brain, liver, lung, and intestine) tested positive for *Protoparvovirus carnivoran1* DNA, while they were negative for CCoV RNA. According to the analysis of the partial VP2 gene sequence obtained from the viral screening, the *Protoparvovirus carnivoran1* viral strain was preliminary typed as the CPV-2b variant based on the VP2 426-Asp residue.

A typical cytopathic effect in the form of cell rounding and detachment of the A-72 cell monolayer was observed at the 4th blind passage, the isolation was confirmed by the PCR assay, and the strain was sequenced (the obtained sequence was identical to the one obtained by the PCR protocol used to amplify the nearly complete CPV-2 sequence). The viral isolate, obtained from this study, was submitted to the Biobanca del Mediterraneo, Italy (www.bbmed.it, accessed on 23 December 2024), to be preserved and long-term stored.

### 3.2. Sequence Analysis

From one of the samples (lung), a 4269 nt long parvoviral sequence, which included the full coding region, was obtained. The nearly complete genome sequence of CPV-2b_IZSSI_2024PA10625 showed the highest nucleotide identities (99.77–99.34%) with CPV-2b strains found in samples collected in 2020 and 2021 from dogs in Turkey (accession numbers: OQ366402, OQ366404, OQ366405, and MW539053) and 99.34% identity with CPV-2b and CPV-2a strains collected in 2015 and 2017, respectively, from dogs in China (accession numbers: MH106699 and MH476590).

The separate analyses of both CPV-2 VP2 and NS1 gene sequences showed high nucleotide identities (99.83−99.72% and 99.70−99.40%, respectively) with CPV-2b strains detected either from dogs and from cats in Egypt and Turkey in 2019 and 2021 or dogs in China in 2011 and in Thailand in 2010 (Supplementary Table S1).

**Figure 1:**
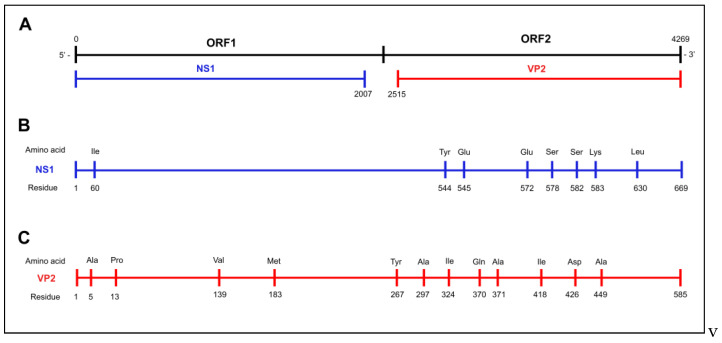
A schematic representation of the CPV-2 genome: the upper lines (**A**) represent the complete encoding nucleotide length (4269 nucleotides) of the genome, excluding the 5′ to the 3′ UTRs, and the relative positions of the NS1 and VP2 genes; the middle ((**B**), in blue) and lower ((**C**), in red) lines represent the relative positions of the described amino-acid changes in the NS1 and VP2 gene sequences, respectively.

Sequence analysis revealed an amino acid pattern which was never observed before in European CPV strains (Table 2 and Table 3). Indeed, specific amino acid residues were identified either in the NS1 gene (582-Ser and 583-Lys) or in the VP2 gene sequence (267-Tyr, 324-Ile, and 440-Ala) shared with CPV-2b strains previously detected most recently in Turkey and Egypt or, less recently, in Thailand and China (Supplementary Table S1). A schematic representation of the CPV-2 genome and the relative positions of the amino-acids described in the NS1 and VP2 gene sequences was depicted in Figure 1.

The comparison of the VP2 gene sequences of CPV-2b strains previously detected in Italy [12] and more recently in Sicily [7] showed nucleotide identities ranging between 99% and 98.2% and amino acid identities ranging between 99.48% and 98.97%. Notably, critical amino acid substitutions (Pro13Ser, Ala371Gly, and Ile418Thr) characteristics of the most recent Italian CPV-2b strains [7,12,13] were not observed in the CPV-2b strain analyzed in this study.

### 3.3. Phylogenetic and Phylogeographic Analyses

Phylogenetic analysis performed on both VP2 and nearly complete genome revealed a strong clustering of the identified strain with sequences from the Middle East, namely Turkey, Egypt, and Iran (Supplementary Figures S1 and S2).

**Figure 2:**
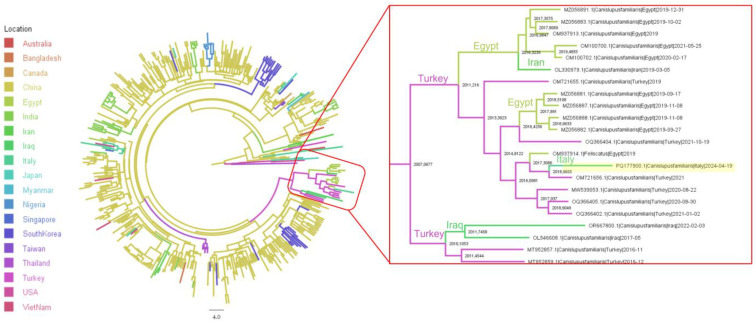
Maximum clade credibility tree of CPV2 strains based on VP2 sequence. Countries where the virus ancestors were estimated to circulate are color-coded. The branch length is scaled in time (years). The clade including the sequence obtained in the present study (highlighted in yellow) is reported in the right insert. Tree nodes are annotated with the estimated year.

The phylogeographic analysis, reconstructing the migration of strain over time, estimated the sampled strain to be part of a clade, originating approximatively in 2007, including strains from the Middle East (i.e., Iran, Iraq, Turkey, and Egypt) stemming from an ancient Asian (likely Chinese) branch (Figure 2). The most likely country of origin for the detected strain was Turkey, with a shared ancestor estimated in 2019. The analysis of the Bayes factor (BF) supporting the migration rates inferred among the locations included in the clade of interest revealed several well-supported connections: China to Iraq (BF = 35.37), China to Turkey (BF = 6.21), Iran to Egypt (BF = 101.34), Iran to Turkey (BF = 84.21), and Turkey to Italy (BF = 14.73).

**Figure 3:**
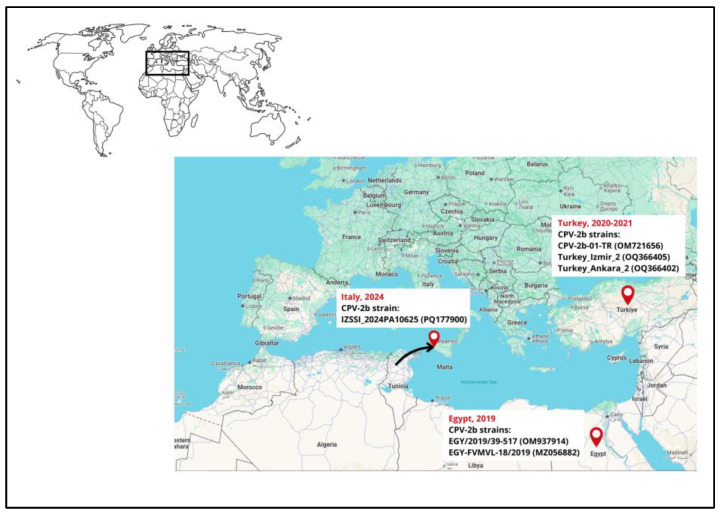
Geographical map of the Mediterranean basin, including year and country of detection of the most related CPV-2b strain to the one described in this study (strain IZSSI_2024PA10625), spreading from Africa to Italy. For each strain, the strain name and the accession number were provided. Each strain refers to the following references: (from Egypt) strain EGY/2019/39-517 [43], EGY-FVMVL-18/2019 [44]; (from Turkey) CPV-2b-O1-TR [45], Turkey_Izmir_2, and Turkey_Ankara_2 [46]. The main image was obtained from Google Earth (Google Landsat / Copernicus Data SIO, NOAA, U.S. Navy, NGA, GEBCOInst. Geogr. NacionalGeoBasis-DE/BKG (©2009) Mapa GISrael).

To map the geographic location and year of detection in the Mediterranean basin, the most related CPV-2b strains described in this study are depicted in Figure 3.

## 4. Discussion

Although vaccination has significantly contributed to the control of clinical cases of parvoviruses in areas where it has been extensively used, canine parvovirus still remains a cause of disease and mortality among juvenile dogs, even after approximately 45 years since its emergence [47]. During this period, most of the studies have been based on CPV-2 detection and typing in specific geographical sites or, less frequently and only more recently, on phylodynamic and phylogeography of detected strains. A wider description is still limited by the temporal discontinuity in the detection or the lack of information from large geographical areas. Therefore, only a few reports or studies have underlined how rapid and long-distance transport of dogs can impact on the spreading dynamics of specific pathogens. Indeed, it has been considered that the ordinary or illegal transport of domestic dogs could act as an underestimated potential risk for the introduction of canine pathogens such as CPV-2 [27,48].

In addition to reported risks related to the conventional routes of dog transport, to the best of our knowledge, this is the first report of a CPV-2 strain, previously never reported in Europe, transported during the Mediterranean maritime refugee crisis. This dog arrived in the Mediterranean coasts of Sicily (Italy) with the owner after recently departing from the North African coast, likely from Tunisia.

Migrations due to factors such as war, famine, and climate change are well-known drivers of pathogens spreading among human populations. However, such dynamics are less known in veterinary medicine, especially for companion animals [49,50,51]. Recently, concerns of rabies spreading have been expressed because of the sudden pet efflux from Ukraine, a high-risk country for rabies, due to the refugee crisis [52]. In fact, the continued socio-economical changes and human migrations could represent alternative routes of animal transport, particularly for pets, which should not be underestimated. Among these, the Mediterranean route of refugees crossing the Central Mediterranean to Europe is constantly monitored.

The detected CPV-2 strain showed an amino acid pattern which was not observed before in European CPV strains, with specific amino acid residues (NS1 gene: 582-Ser and 583-Lys; VP2 gene: 267-Tyr, 324-Ile, and 440-Ala). These specific amino acids were shared with CPV-2b strains detected in 2019 and 2021 in Turkey, where this variant is the most detected to date [45,46], and in 2019 from a cat in Egypt [43,44], thus proving the circulation of this variant in the Middle East area. Interestingly, a 100% amino acid identity was observed for the detected strain with the partial VP2 sequence of the CPV-2b strain Tr-Fox/159 (accession number: MW259074), which was first detected in Turkey from the feces of a red fox and collected in 2015, and for which a transmission bridge of CPV-2 between domestic dogs and wild animals or vice versa has been hypothesized [53]. In turn, the origin or co-evolution with strains of Asian origin was proposed for these CPV-2 strains from Turkey and Egypt [44,45], which agrees with present study findings, where a close relationship with strains detected in 2010 and 2011 in Thailand and China, respectively [54,55], was observed.

Although CPV-2 variant is the least prevalent in the continental Italy [12], with its frequency halving during the last years in Sicily [7]—the region where this dog arrived, the current literature is consistent with the circulation of CPV-2b strains with specific amino acid changes (Pro13Ser, Ala371Gly, and Ile418Thr) in Italy. The amino acid patterns of the described strain, referring to these characteristic amino acid residues, demonstrated a clear divergence (13-Pro, 371-Ala, and 418-Ile) from Italian CPV-2b strains, in addition to other strain specific residues, as discussed above. Although the spread of this strain in Italy was likely prevented by the prompt isolation and death of the animals, the large-scale spreading potential is a consistent threat. Further large-scale molecular studies are therefore necessary to evaluate whether a progressive spread has occurred among host populations in Italy or whether this even represents an occasional episode without diffusion. Indeed, in 2017, a CPV-2c strain originating from Thailand was introduced into Southern Italy with a dog [26], and following studies evidenced its rapid spread in Italy [27] and its rapid replacement of regional circulating CPV-2c strains [7]. Other cases of introduction of this CPV-2c variant of Asian origin, again introduced through dogs from East European countries to Northeastern Italy, were also documented [23]. The evidence of such a rapidly evolving epidemiological scenario underscores the need for constant evaluation of circulating strains to promptly identify any changes and evaluate their relevance.

For the latter CPV-2c variant, concerns have been raised for the effectiveness of the current vaccines. These concerns were mostly corroborated by the speculative evidence of some specific amino acid changes, considered as potentially responsible for vaccine failure [56]. The CPV-2b strain described in this study showed the same amino acids at specific residues in the VP2 gene (267-Tyr, 324-Ile, and 440-Ala). In any case, specific studies and evaluations on cross-protection have not been already performed, while the ability of classical or recombinant commercially available vaccines towards the classical CPV-2 variants has been proved [57,58,59,60]. In this study, no definitive information on the vaccination status of the sampled dogs was available, which prevents any firm conclusions from being drawn regarding vaccine protection. This lack of data contributes to the overall uncertainty and highlights the need for dedicated studies to better understand the determinants of cross-protection. As previously suggested [7], most recent viral strains showed these amino acid residues could be suggested for additional future studies, aiming to address the specific uncertainties observed regarding immune protection of vaccines.

CPV-2 variants displaying these VP2-267Tyr, -324Ile, and -440Ala residues are considered immune escape mutants and associated to vaccine failure [56], and these amino acidic residues are considered as important for virus host adaptation, host range, and antigenicity [61]. Limited information on the NS1 gene sequence of Asian, Turkish, or Egyptian CPV-2 strains is available, thus preventing further structural or epidemiological evaluations.

The clustering observed in the phylogenetic tree reconstruction highlighted a close relationship with strains of Middle Eastern origin, which are themselves linked to Asian strains. This suggests potential contact between these regions, followed by subsequent migration towards Italy. This hypothesis was formally confirmed by the phylogeographic analysis, which demonstrated the inclusion of the sequence under investigation within a cluster containing Middle Eastern-origin sequences, specifically those of Iranian, Iraqi, Turkish, and Egyptian origin. Considering that these countries may be part of migration routes, particularly from Asian regions where the viral clade in question appears to have originated, it is possible to speculate about a progressive westward dispersion of the strains towards North Africa over the years, likely in the last decade.

It is noteworthy that this route mirrors several migratory paths of people fleeing war-torn areas or economic hardship. The most recent common ancestor, estimated about five years ago, and its Turkish origin (not the most western country in the hypothesized path) could partially refute the direct role of migratory routes in shaping viral epidemiology. However, the limited number of sequences available from this region and North African regions along the migratory route might obscure a closer connection to migratory pathways, preventing a precise assessment of their role. An alternative hypothesis is that the virus spread in these regions through other mechanisms and socio-cultural contacts, with the animal becoming infected by locally circulating viruses just before transportation. Regardless of the specific mechanism, the relevance of these human migrations in influencing viral spread is evident. Whether this represents a consistent factor over time, i.e., a gradual migration from Asia to neighboring North African regions, or consists of truly sporadic events remains a subject for further investigation.

A more consistent and conclusive route was not possible to be determined; nonetheless, an uncommon route of pathogen introduction to Italy, posing a serious threat to dogs, was revealed. Further studies are then necessary to define any additional geographical spreading patterns in North African countries or any further spread in Europe. Moreover, continuous monitoring of conventional or unconventional routes of dog transport is necessary to provide early warnings for threats following viral introduction, to apply necessary biosecurity measures and prevent their spread. More comprehensive and updated epidemiological studies of CPV-2 in the countries of the Mediterranean basin area could be beneficial to re-evaluate the viral dynamics.

## Figures and Tables

**Table 1:** Primers used for the CPV-2 screening PCR assay and sequencing.

Assay	Primer ^b^	Target	Sequence (5x-3′)	Position ^a^	Amplicon Size (bp)	Reference
PCR for detection ^c^	VP2-850-Forward	VP2 gene	GAGCATTGGGCTTACCA	3633–3649	700	[30]
	VP2-1550-Reverse		GCAGATGCATCAGGATC	4316–4332		
Genome sequencing ^d^	NS-Fext	ORF1	GACCGTTACTGACATTCGCTTC	206–227	2255	[17]
	NS-Rext		GAAGGGTTAGTTGGTTCTCC	2441–2460		
	2161F	ORF2	TTGGCGTTACTCACAAAGACGTGC	2160–2183	2788	
	4823R		ACCAACCACCCACACCATAACAAC	4924–4947		
ORF1 sequencing internal primers ^e^	NS-Fint		GTTGAAACCACAGTGACGACAG	1055–1076		
	NS-Rint		CATCATCCAGTCTTCAGGTG	1167–1186		
ORF2 sequencing internal primers ^e^	3475R		GTTGGTGTGCCACTAGTTCCAGTA	3451–3474		
	R2		TTTTGAATCCAATCTCCTTCTGGAT	4011–4035		

^a^ Nucleotide positions refer to the prototype CPV strain CPV-N (USA, 1978; accession number: M19296). ^b^ Primers used for the CPV-2 detection indicated by the letter ^c^ (targeting the partial VP2 gene sequence) or for the CPV-2 full-length genome sequence amplification indicated by the letter ^d^. ^e^ Internal primers used for sequencing.

**Table 2:** Amino acid variations in the NS1 sequence of the analyzed CPV-2b strain.

Variant	Strain	Country	Year	Acc. Nr.	60	544	545	572	578	582	583	630
CPV-2 ^1^	CPV-b	USA	1978	M38245	I	Y	E	E	G	L	E	L
CPV-2a ^1^	43-97	Italy	1997	MF177224	-	F	-	-	-	-	-	-
CPV-2b ^1^	1-99	Italy	1999	MF177226	-	F	-	K	-	-	-	-
CPV-2b ^1,2^	CPV-2b_IZSSI_2022PA2773	Italy ^4^	2022	ON677437	V	F	V	-	-	-	-	P
CPV-2b ^1^	Turkey_Ankara_2	Turkey	2021	OQ366402	-	-	-	-	-	S	K	-
CPV-2c ^1^	485-09	Italy	2009	MF177228	-	-	-	-	-	-	-	-
CPV-2c ^1,2^	CPV_IZSSI_2743_17	Italy ^4^	2017	MF510157	V	F	V	-	-	-	-	P
CPV-2a ^1^	CPV/CN/LN1/2014	China ^4^	2014	KR002800	V	F	V	-	-	-	-	P
CPV-2b ^1^	CPV-BJL2	China ^4^	2015	MH106699	-	-	-	K	-	-	-	-
CPV-2c ^1^	Canine/China/12/2017	China ^4^	2017	MH476581	V	F	V	-	-	-	-	P
**CPV-2b ^3^**	**CPV-2b_IZSSI_2024PA10625**	**Italy**	**2024**	**PQ177900**	**I**	**Y**	**E**	**E**	**S**	**S**	**K**	**L**

^1^ Reference strain; ^2^ collected in the same region as the strain from this study; ^3^ strain analyzed in this study; ^4^ strain belonging to the Asian CPV-2 lineage; “-” the same amino acid as in the first row. According to IUPAC codes: E, Glu, Glutamic Acid; F, Phe, Phenylalanine; G: Gly, Glycine; I: Ile, Isoleucine; K: Lys, Lysine; L: Leu, Leucine; P: Pro, Proline; S: Ser, Serine; V: Val, Valine; Y: Tyr, Tyrosine. The last row was bolded to highlight data and features of the analyzed strain sequence.

**Table 3:** Amino acid variations in the VP2 sequence of the analyzed CPV-2b strain.

Variant	Strain	Country	Year	Acc. Nr.	5	13	139	183	267	297	324	370	371	418	426	440
CPV-2	CPV-b	USA	1978	M38245 ^1^	A	P	V	M	F	S	Y	Q	A	I	N	T
CPV-2a	43-97	Italy	1997	MF177224 ^1^	-	-	-	-	-	A	-	-	-	-	-	A
	IZSSI_2019PA10949	Italy	2019	OR463522 ^1,2^	-	-	-	-	-	A	L	-	-	-	-	
	IZSSI_2019PA5124id436	Italy	2019	OR463514 ^1,2^	-	-	-	-	-	A	L	-	-	-	-	A
	IZSSI_2020CT1227	Italy	2019	OR463518 ^1,2^	-	-	I	-	-	A	I	-	-	-	-	-
CPV-2b	1-99	Italy	1999	MF177226 ^1^	-	-	-	-	-	-	-	-	-	-	D	-
	IZSSI_2019PA26796	Italy	2019	OR463533 ^1,2^	-	S	-	-	-	A	-	-	G	T	D	-
	IZSSI_2019RG11304	Italy	2019	OR463563 ^1,2^	-	-	-	-	-	A	-	-	G	T	D	-
	IZSSI_2022PA15678idMeF	Italy ^4^	2022	OR463607 ^1,2^	G	-	-	-	Y	A	I	R	-	-	D	-
CPV-2b	CPV-2b_IZSSI_2022PA2773	Italy ^4^	2022	ON677437 ^1,2^	G	-	-	-	Y	A	I	R	-	-	D	-
CPV-2b	Turkey_Ankara_2	Turkey	2021	OQ366402 ^1^	-	-	-	-	Y	A	I	-	-	-	D	A
CPV-2c	485-09	Italy	2009	MF177228 ^1^	-	-	-	-	-	A	-	-	-	-	E	-
	IZSSI_2019RG7696	Italy	2019	OR463566 ^1,2^	-	-	-	-	-	A	-	-	-	-	E	-
	IZSSI_2019PA30397	Italy	2019	OR463579 ^1,2^	-	-	I	-	-	A	-	-	-	-	E	-
	IZSSI_2019RG11305	Italy	2019	OR463565 ^1,2^	-	S	-	-	-	A	-	-	-	-	E	-
	IZSSI_2019PA28001	Italy ^4^	2019	OR463616 ^1,2^	-	-	-	-	Y	A	I	R	-	-	E	-
	IZSSI_2020PA53415	Italy ^4^	2020	OR463658 ^1,2^	G	-	-	-	Y	A	I	R	-	-	E	-
	IZSSI_2021PA43108idAki	Italy ^4^	2021	OR463654 ^1,2^	-	-	-	-	Y	A	I	R	-	-	E	A
	IZSSI_2022PA19220idC1	Italy ^4^	2022	OR463610 ^1,2^	-	-	-	I	Y	A	I	R	-	-	E	-
CPV-2c	CPV_IZSSI_2743_17	Italy ^4^	2017	MF510157 ^1,2^	G	-	-	-	Y	A	I	R	-	-	E	-
CPV-2a	CPV/CN/LN1/2014	China ^4^	2014	KR002800 ^1^	-	-	-	-	Y	A	I	-	-	-	-	A
CPV-2b	CPV-BJL2	China ^4^	2015	MH106699 ^1^	-	-	-	-	Y	A	I	-	-	-	D	A
CPV-2c	Canine/China/12/2017	China ^4^	2017	MH476581 ^1^	G	-	-	-	Y	A	I	R	-	-	E	-
**CPV-2b**	**CPV-2b_IZSSI_2024PA10625**	**Italy**	**2024**	**PQ177900 ^3^**	**A**	**P**	**V**	**M**	**Y**	**A**	**I**	**Q**	**A**	**I**	**D**	**A**

^1^ Reference strain; ^2^ collected in the same region as the strain from this study; ^3^ strain analyzed in this study; ^4^ strain belonging to the Asian CPV-2 lineage; “-” the same amino acid as in the first row. According to IUPAC codes: A: Ala, Alanine; D: Asp, Aspartic acid; E: Glu, Glutamic acid; F: Phe, Phenylalanine; G: Gly, Glycine; I: Ile, Isoleucine; L: Leu, Leucine; M: Met, Methionine; N: Asn, Asparagine; P: Pro, Proline; Q: Gln, Glutamine; R: Arg, Arginine; S: Ser, Serine; T: Thr, Threonine; V: Val, Valine; Y: Tyr, Tyrosine. The last row was bolded to highlight data and features of the analyzed strain sequence.

## Data Availability

The sequence data obtained in this study have been submitted to GenBank database under accession number PQ177900 (isolate IZSSI_2024PA10625).

## Supplementary Materials

The following supporting information can be downloaded at: https://www.mdpi.com/article/10.3390/pathogens14020108/s1, Table S1: Nucleotide identities with VP2 and NS1 gene sequences of CPV-2b strains; Figure S1: Maximum likelihood phylogenetic tree of CPV2 strains based on VP2 sequence. The clade including the sequence obtained in the present study (highlighted in yellow) is magnified in the right insert. Tree nodes are annotated with the respective bootstrap support; Figure S2: Maximum likelihood phylogenetic tree of CPV2 strains based on the complete genome sequence. The clade including the sequence obtained in the present study (highlighted in yellow) is magnified in the right insert. Tree nodes are annotated with the respective bootstrap support.
