# Supplementary material for: Introduction of a Divergent Canine Parvovirus Type 2b Strain with a Dog in Sicily, Southern Italy, Through the Mediterranean Sea Route to Europe
Source: Pathogens. 2025 Jan 23;14(2):108. doi: 10.3390/pathogens14020108 (PMC11857852; doi:10.3390/pathogens14020108)
Supplement: Supplementary file 1 [file pathogens-14-00108-s001.zip › pathogens-3416637-Table S1.pdf]

Supplementary Material – Table S1. Nucleotide identities with VP2 and NS1 gene sequences of CPV-2b strains.

Nucleotide identity	Acc.nr.	Isolate/strain name	Host	Country	Year	Reference
		**VP2 gene**				
99.83%	OM937914	EGY/2019/39-517	Cat	Egypt	2019	[46]
	OM721656	CPV-2b-O1-TR	Dog	Turkey	2021	[52]
99.77%	OQ366405	Turkey_Izmir_2	Dog	Turkey	2020	[45]
	OQ366402	Turkey_Ankara_2	Dog	Turkey	2021	[45]
	KP715703	CPV-VT86	Dog	Thailand	2010	[55]
	MW539053	I1	Dog	Turkey	2020	[45]
99.72%	KP715712	CPV-VT123	Dog	Thailand	2010	[55]
	KP715700	CPV-VT75	Dog	Thailand	2010	[55]
	KP715690	CPV-VT28	Dog	Thailand	2010	[55]
	KP715689	CPV-VT18	Dog	Thailand	2010	[55]
	MZ056882	EGY-FVMVL-18/2019	Dog	Egypt	2019	[43]
		**NS1 gene**				
99.70%	OQ366402	Turkey_Ankara_2	Dog	Turkey	2021	[45]
99.55%	OQ366405	Turkey_Izmir_2	Dog	Turkey	2020	[45]
99.40%	OR066202	TR.CPV.SAMSUN.03	Dog	Turkey	2020	Unpublished
	OR066200	TR.CPV.SAMSUN.01	Dog	Turkey	2022	Unpublished
99.35%	OR066203	TR.CPV.SAMSUN.04	Dog	Turkey	2022	Unpublished
99.30%	OR066204	TR.CPV.SAMSUN.05	Dog	Turkey	2022	Unpublished
	OQ366404	Turkey_Sanliurfa_3	Dog	Turkey	2021	[45]
	JQ268283	CPV-LZ1	Dog	China	2011	[53]
