# Supplementary figures and images for: Introduction of a Divergent Canine Parvovirus Type 2b Strain with a Dog in Sicily, Southern Italy, Through the Mediterranean Sea Route to Europe
Source: Pathogens. 2025 Jan 23;14(2):108. doi: 10.3390/pathogens14020108 (PMC11857852; doi:10.3390/pathogens14020108)

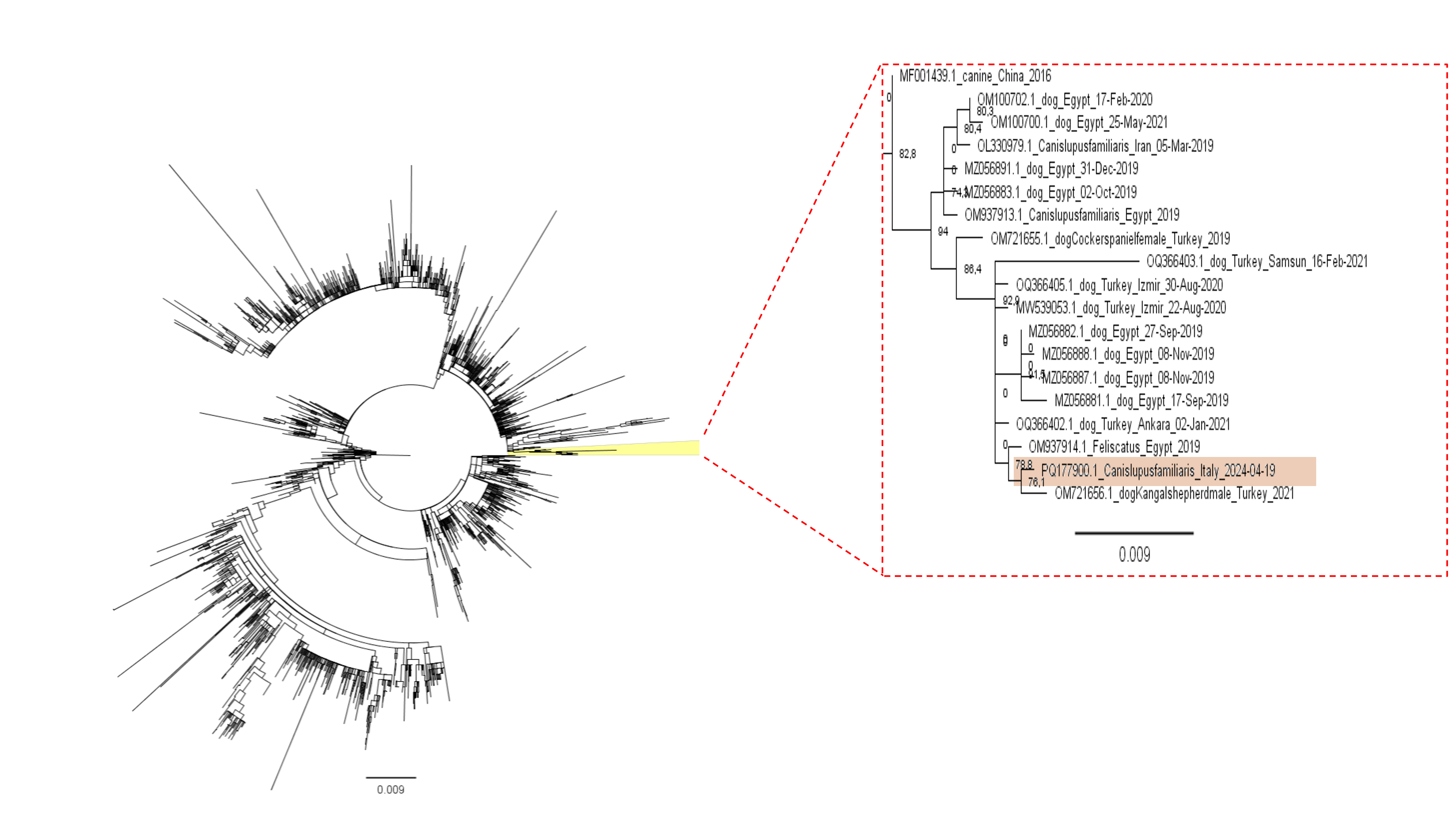

Supplement: Supplementary file 1 [file pathogens-14-00108-s001.zip › Supplementary Material - Figure S1.png]

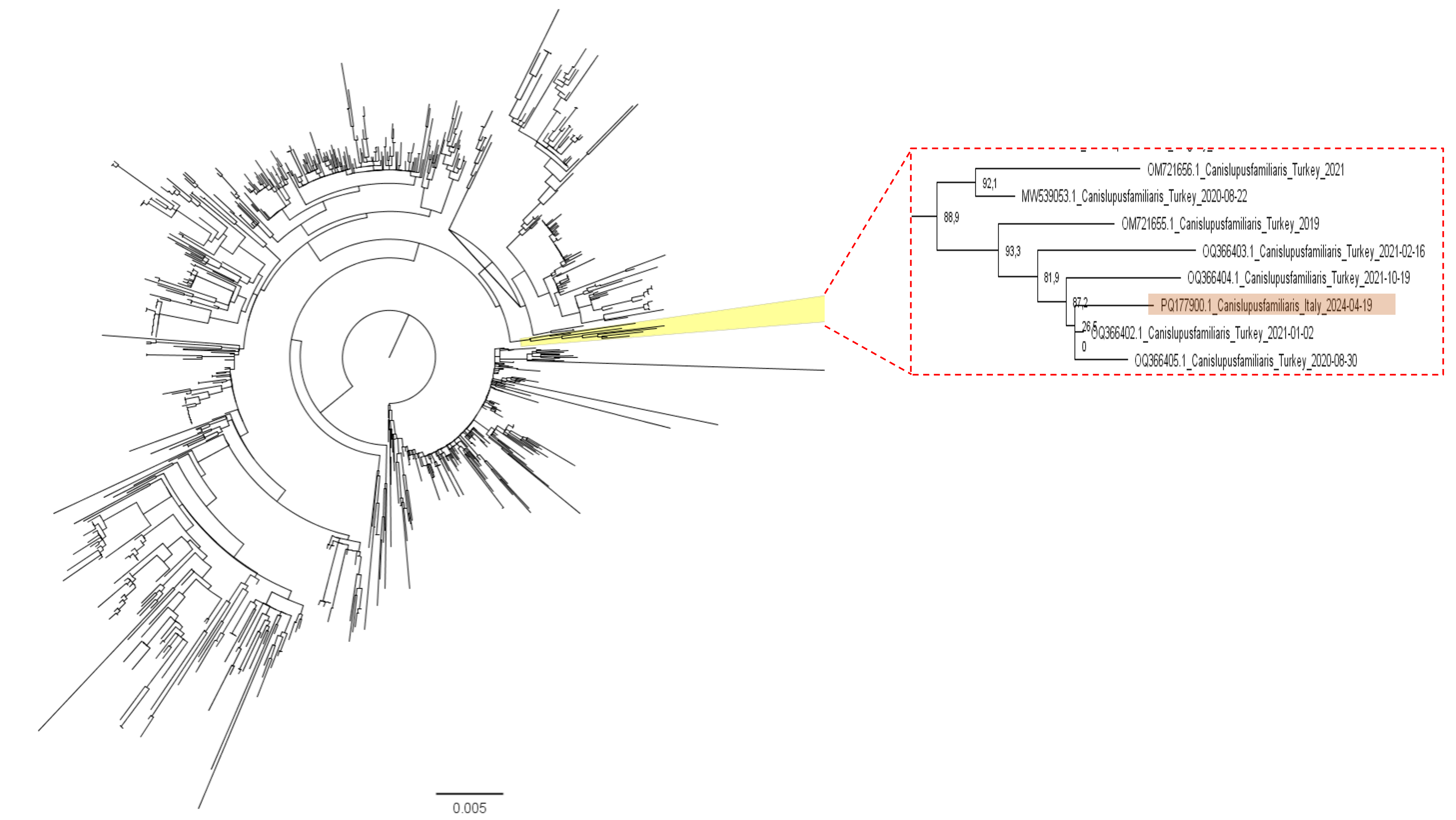

Supplement: Supplementary file 1 [file pathogens-14-00108-s001.zip › Supplementary Material - Figure S2.png]
